# The Last Decade of Optically Active α-Aminophosphonates

**DOI:** 10.3390/molecules28166150

**Published:** 2023-08-20

**Authors:** Petra R. Varga, György Keglevich

**Affiliations:** Department of Organic Chemistry and Technology, Budapest University of Technology and Economics, 1521 Budapest, Hungary; varga.petra.regina@vbk.bme.hu

**Keywords:** optically active α-aminophosphonates, asymmetric syntheses, organocatalysts, Kabachnik–Fields reactions, aza-Pudovik reactions, enantioselective hydrogenations, green syntheses

## Abstract

α-Aminophosphonates and related compounds are important due to their real and potential biological activity. α-Aminophosphonates may be prepared by the Kabachnik–Fields condensation of oxo compounds, amines and dialkyl phosphites, or by the aza-Pudovik addition of the same P-reagents to imines. In this review, the methods that allow for the synthesis of α-aminophosphonates with optical activity are surveyed. On the one hand, optically active catalysts or ligands may induce enantioselectivity during the Kabachnik–Fields reaction. On the other hand, asymmetric catalysis during the aza-Pudovik reaction, or hydrogenations of iminophosphonates, may prove to be a useful tool. Lastly yet importantly, it is possible to start from optically active reagents that may be associated with diastereoselectivity. The “green” aspects of the different syntheses are also considered.

## 1. Introduction

α-Aminophosphonates form a representative group within phosphonates due to their potential biological activity, enabling them to be used in the pharmaceutical industry [1]. The biological activity is related to the enzyme inhibitory properties of the compounds under discussion. The biological activity includes anticancer and anti-HIV effects, among others. The basic methods for the synthesis of α-aminophosphonates are the Kabachnik–Fields condensation and the aza-Pudovik reaction [2]. The phospha-Mannich condensation involves the reaction of an oxo compound, such as an aldehyde or ketone, a primary and secondary amine, and dialkyl phosphite, while the Pudovik approach utilizes the addition of diakyl phosphites to the double bond of imines. As a matter of fact, the imines formed from the oxo compound and the primary amine may be the intermediate of the Kabachnik–Fields reaction.

It is a challenge to run the syntheses of α-aminophosphonates in an enantioselective manner. The stereoselective syntheses of α-aminophosphonic derivatives were summarized by Ordóñez and colleagues [3], and Palacios et al. [4]. The phospha-Mannich condensation of an oxo compound, amine and >P(O)H reagent may be carried out in the presence of an optically active catalyst or additive to obtain the corresponding product as a predominant enantiomer. The most relevant method for the preparation of optically active α-aminophosphonates is the enantioselective addition of dialkyl phosphites to the C=N unit of imines [5,6,7,8,9,10]. This is promoted by chiral catalysts or additives. The asymmetric hydrogenation of iminophosphonates is also an attractive method of choice. Lastly yet importantly, the Kabachnik–Fields reactions were performed using optically active amines, aldehydes or phosphites as the starting materials [8,9,10,11].

## 2. Synthetic Approaches for Optically Active α-Aminophosphonates

### 2.1. Enantioselective Kabachnik–Fields Reactions

**Scheme 1:**
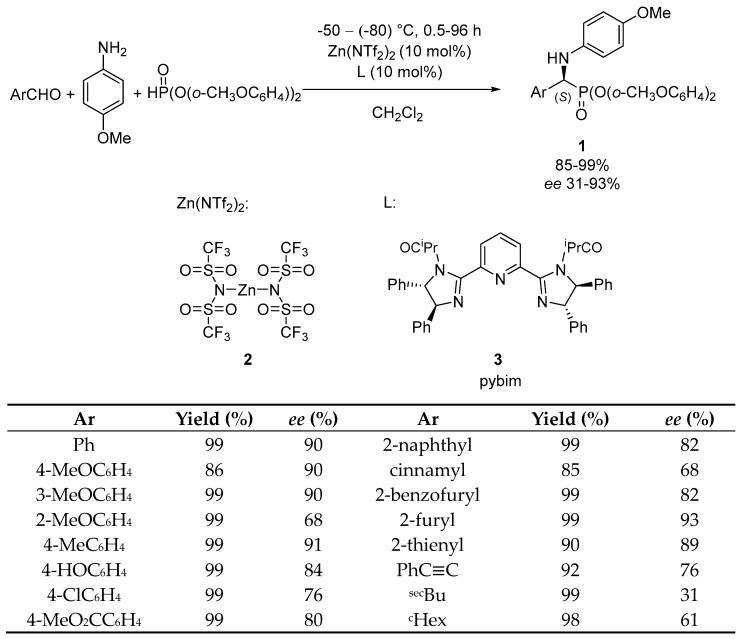
Enantioselective synthesis of α-aminophosphonates (**1**) using zinc bis(trifluoromethylsulfonyl)imide (**2**, **3**) as the catalyst.

An enantioselective three-component Kabachnik–Fields reaction was elaborated to provide (*S*)-α-aminophosphonates (**1**). The method comprised zinc bis(trifluoromethylsulfonyl)imide (**2**) as the catalyst, along with an optically active ligand (1,3-bis(imidazolin-2-yl)pyridine: pybim) (**3**) in dichloromethan as the solvent at −50–−80 °C. The enantiomeric purity of the resulting aminophosphonates (**1**) depended on the substituent in the aromatic ring (Scheme 1) [12]. The highest ee values (90–93%) were obtained by starting from 3-methoxy- or 4-methylbenzaldehyde or 2-furylaldehyde.

**Scheme 2:**
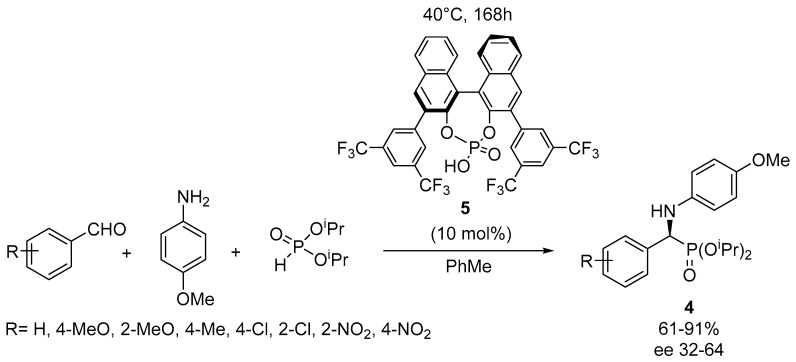
Three-component asymmetric Kabachnik–Fields reaction using optically active phosphinic acid derivative (**5**) as the catalyst.

The condensation of benzaldehydes, para-aminoanizole and diisopropyl phosphite gave the corresponding aminophosphonates (**4**) under mild conditions, and when using an optically active phosphinic acid derivative (**5**) as the catalyst (Scheme 2) [13].

**Scheme 3:**
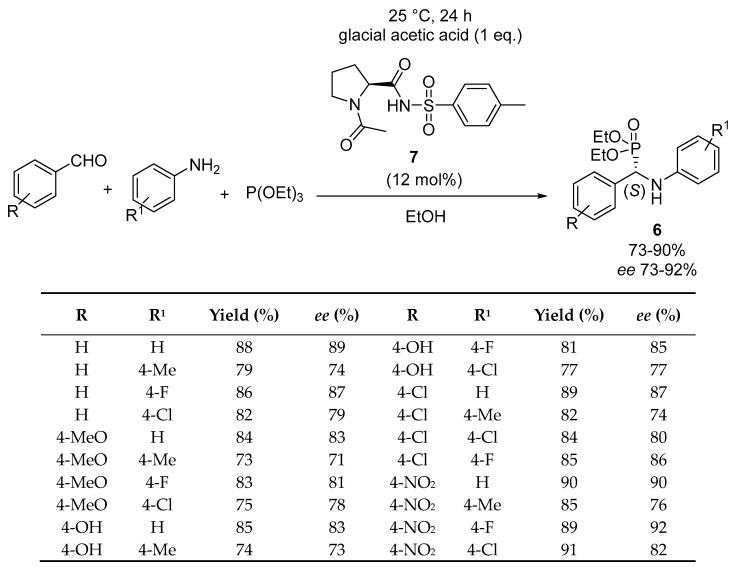
Synthesis of α-aminophosphonates (**6**) using a pyrrolidine-based organocatalyst (**7**).

A series of optically active α-aminophosphonate derivatives (**6**) was synthesized under mild conditions by applying substituted benzaldehydes, aniline derivatives and, in this case, triethyl phosphite and a chiral pyrrolidine-based organocatalyst (**7**). The yields fell in the range of 71–90%, and the enantiomeric purity was 73–92% (Scheme 3) [14]. The use of triethyl phosphite instead of diethyl phosphite is not advantageous due to the smell and atom efficiency of the condensation reaction.

**Scheme 4:**
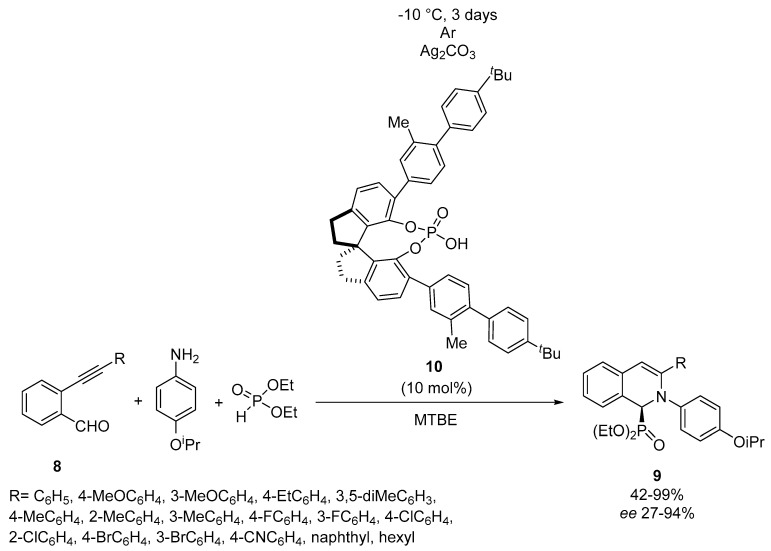
Three-component reaction of 2-alkynylbenzaldehydes (**8**), an aniline derivative, and diethyl phosphite to provide α-aminophosphonates (**9**).

The three-component reaction of 2-alkynylbenzaldehydes (**8**), an aniline derivative, and diethyl phosphite produced cyclic α-aminophosphonates (**9**) in the presence of silver carbonate, and an optically active spirocyclic phosphoric acid (**10**) as the catalyst. The yields and enantiomeric purities were variable (Scheme 4) [15].

In the above sub-chapter, enantioselective Kabachnik–Fields reactions as described in the literature were summarized. The methods applied different optically active catalysts under a wide variety of reaction conditions. The efficiency of the catalysts depended on the nature of the reaction models. The products were obtained in good yields and in variable, 31–94% enantioselectivities. These catalysts are not simple, and in most cases, their cost is considerable, which constitutes a shortcoming.

### 2.2. Optically Active α-Aminophosphonate Derivatives Provided by the Aza-Pudovik Reaction

**Scheme 5:**
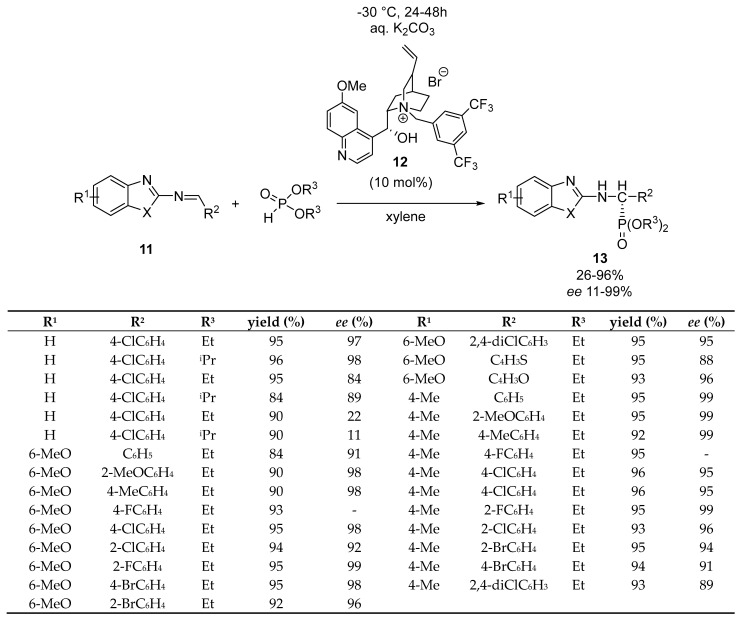
Asymmetric addition of dialkyl phosphites to imines (**11**) in the presence of a chiral PTC catalyst (**12**).

A highly enantioselective nucleophilic addition of dialkyl phosphites to imines, which was (**11**) catalyzed by a chiral cinchona-based phase transfer catalyst (**12**), was developed. Phase transfer catalysis is an up-to-date and “green” approach. The hydrophosphonylation took place in good to high yields and enantioselectivities (Scheme 5) [16].

**Scheme 6:**
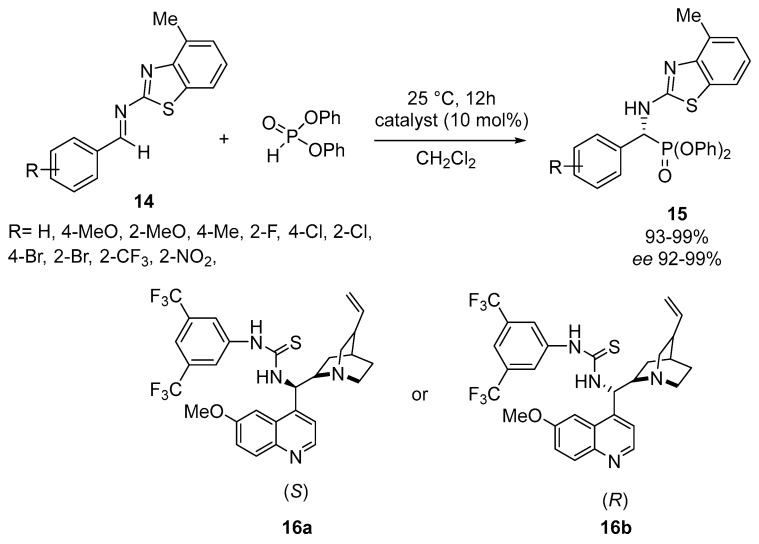
Asymmetric synthesis of biologically active α-aminophosphonates (**15**).

α-Aminophosphonates containing an *N*-benzothiazole moiety (**15**) were synthesized from imine **14** in excellent yields, and enantiomeric purities were synthesized using chiral thiourea-coupled cinchona organocatalysts (**16a** or **16b**). These derivatives displayed activity against cucumber mozaik virus (Scheme 6) [17].

**Scheme 7:**
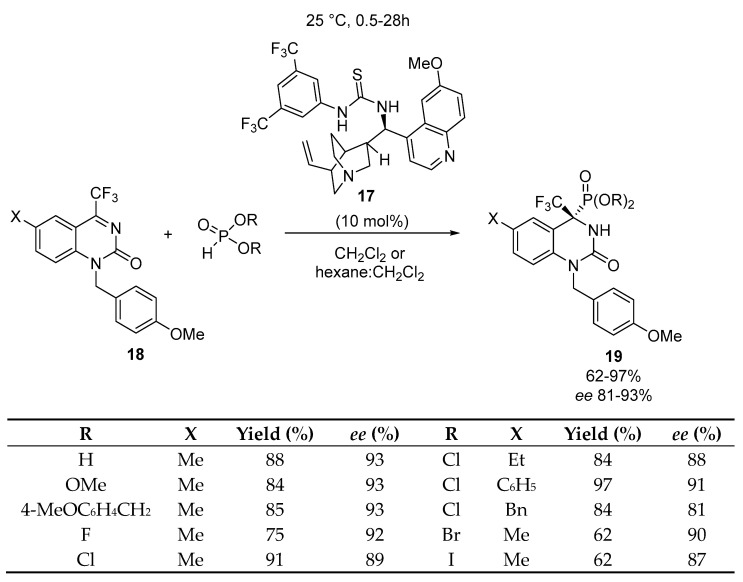
Cinchona (**17**)-catalyzed enantioselective aza-Pudovik reaction.

Cinchona-catalyzed (**17**) enantioselective hydrophosphonylation of trifluoromethyl-substituted quinazolinone derivatives (**18**) afforded the corresponding α-aminophosphonates (**19**) in good yields, and gave enantiomeric purities (Scheme 7) [18].

Other heterocyclic α-aminophosphonic species, such as quinoxalin derivatives, were also prepared by the aza-Pudovik approach [19].

**Scheme 8:**
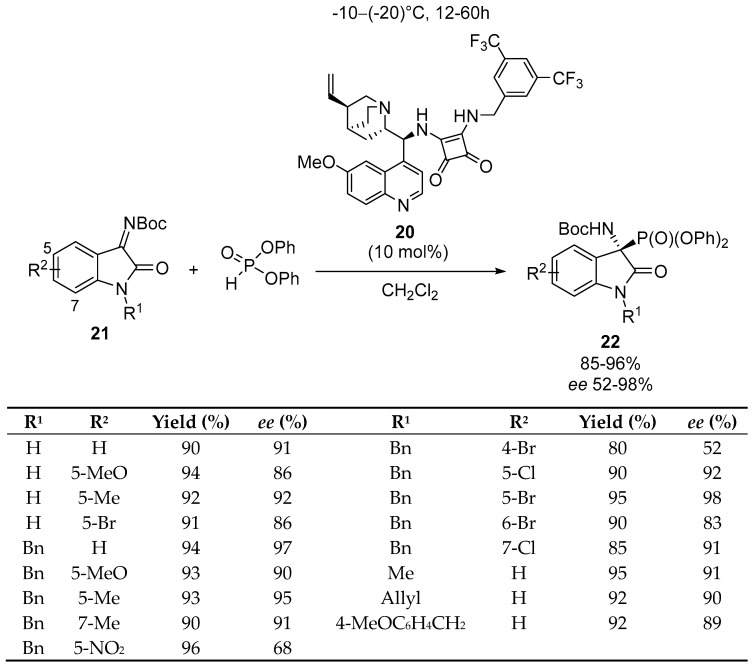
Quinine-squaramide (**20**)-catalyzed enantioselective addition of diphenyl phosphite to ketimines (**21**).

Quinine-squaramide (**20**) catalyzed the enantioselective addition of diphenyl phosphite to imines (**21)** to give the corresponding optically active adducts (**22**) in good yields, and in most cases, high enantioselectivities (Scheme 8) [20].

**Scheme 9:**
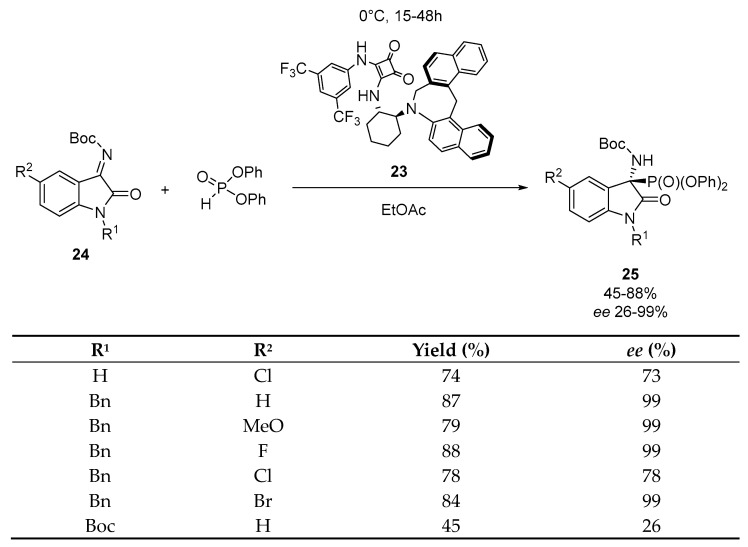
Synthesis of α-aminophosphonates (**25**) by the aza-Pudovik reaction of ketimines (**24**).

The chiral binaphthyl-modified squaramide (**23**)-catalyzed enantioselective addition of diphenyl phosphite to imines (**24**) was also described. This method afforded the corresponding aminophosphonates (**25**) in high yields with excellent (up to 99%) enantioselectivities (Scheme 9) [21].

**Scheme 10:**
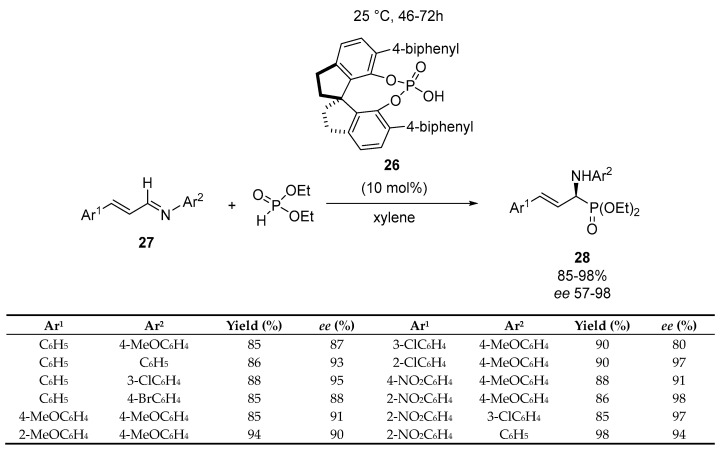
**A** SPINOL (**26**)-based chiral phosphoric acid-catalyzed aza-Pudovik reaction.

A SPINOL-based phosphoric acid-catalyzed (**26**) enantioselective reaction of cinnamaldehyde-derived aldimines (**27**) and diethyl phosphite gave the corresponding α-aminophosphonate derivatives (**28**) under mild conditions in 85–98 yields (Scheme 10) [22].

**Scheme 11:**
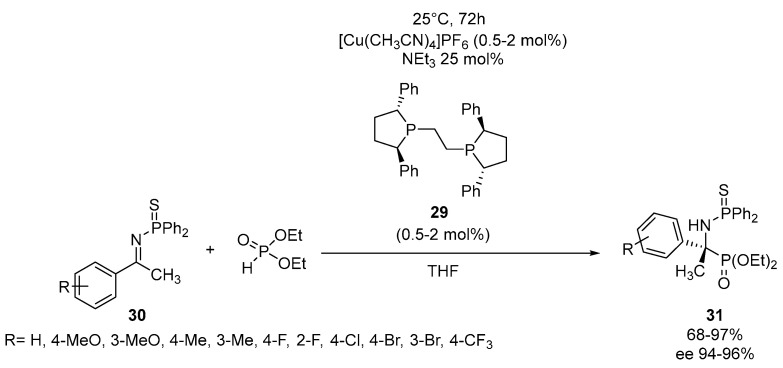
Asymmetric hydrophosphonylation of iminophosphonates (**30**).

An (*R*,*R*)-Ph-BPE-catalyzed (**29**) asymmetric hydrophosphonylation of *N*-thiophosphinoyl imines (**30**) with dialkyl phosphites was performed at room temperature (Scheme 11) [23]. The method gave the corresponding α-aminophosphonates (**31**) in 68–97% yields and 94–96% enantiomeric purities.

In this sub-chapter, enantioselective aza-Pudovik addition reactions were summarized. The presented chiral catalyst-promoted methods gave the corresponding products in variable yields and enantiomeric purities. The typical catalysts are the cinchona alkaloids, or their modified versions. There were no data released on the re-circulation of the organocatalysts. The aza-Pudovik addition is an attractive approach due to the mild conditions required, and the 100% atomic efficiency.

### 2.3. Synthesis of Enantiopure α-Aminophosphonates by the Asymmetric Hydrogenation of Iminophosphonates

**Scheme 12:**
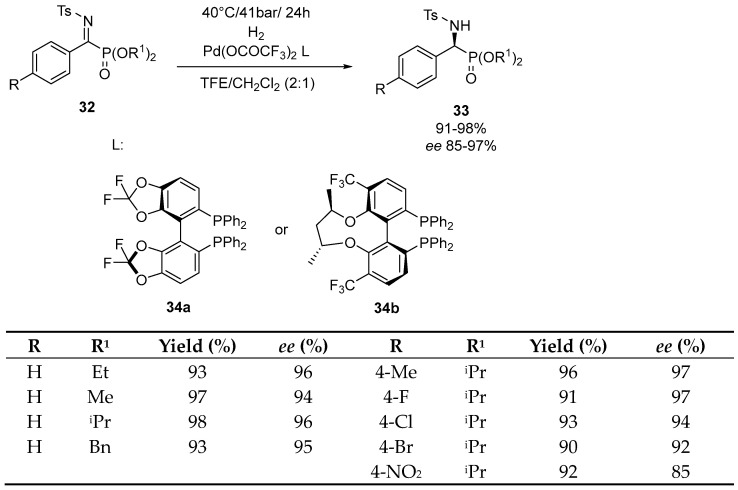
Synthesis of (*S*)-α-phenyl-α-amino-methylphosphonates (**33**).

The palladium-catalyzed asymmetric hydrogenation of α-iminophosphonates (**32**) performed at 40 °C for 24 h in a solvent mixture afforded the corresponding α-aminophosphonates (**33**) in 91–98% yields and 85–97% enantiomeric purities. (*R*)-Difluorophos or its analogue (**34a** or **34b**) served as the ligand for Pd (Scheme 12) [24,25].

**Scheme 13:**
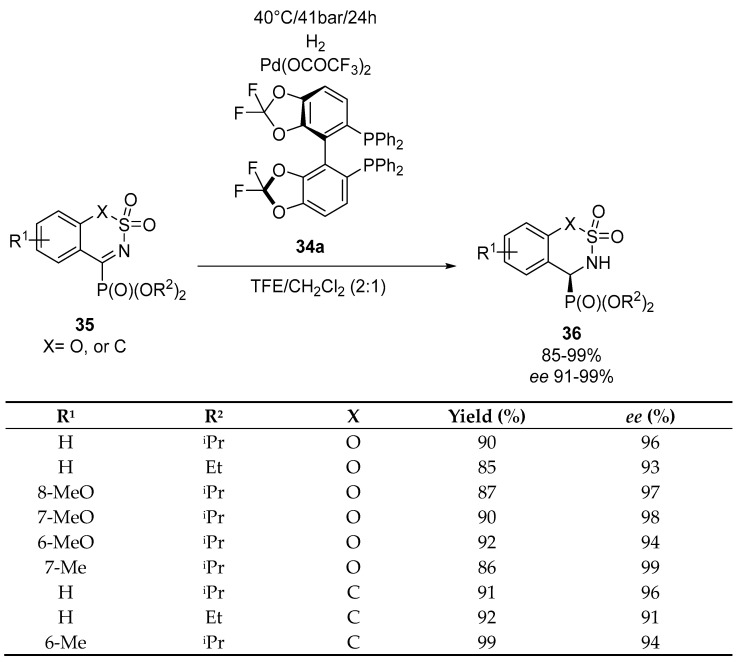
Pd-catalyzed asymmetric hydrogenation of ring α-iminophosphonates (**36**).

A Pd/(*R*)-difluorophos (**34a**)-catalyzed hydrogenation was also described as affording cyclic sulfonamido derivatives (**36**) (Scheme 13) [25].

**Scheme 14:**
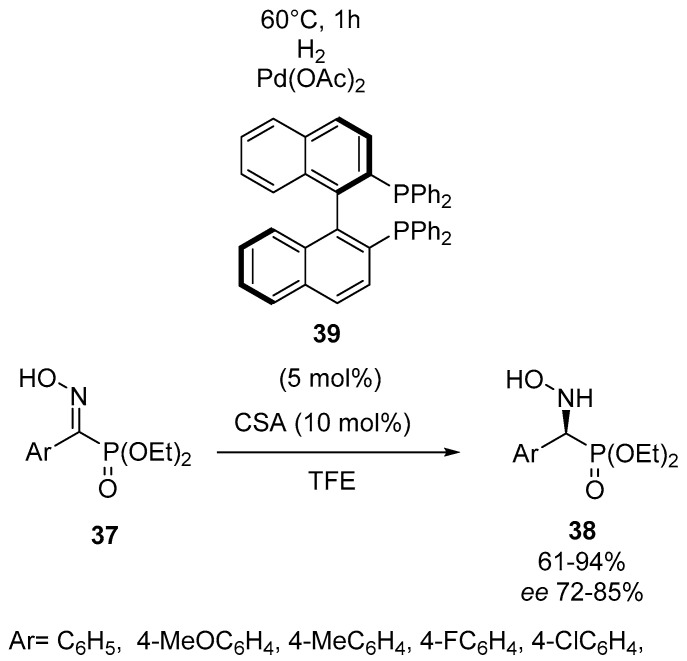
A Pd-catalyzed asymmetric hydrogenation of hydroxy-iminophosphonates (**37**).

Asymmetric hydrogenation of hydroxy-iminophosphonates (**37**) gave the corresponding hydroxy-aminophosphonates (**38**) in up to 90% *ee* using catalytic amounts of palladium(II)acetate, together with an (*R*)-BINAP (**39**) ligand in 2,2,2-trifluoroethanol (TFE). (1*S*)-(+)-10-Camphorsulfonic acid (CSA) served as a Brønsted acidic activator (Scheme 14) [26].

**Scheme 15:**
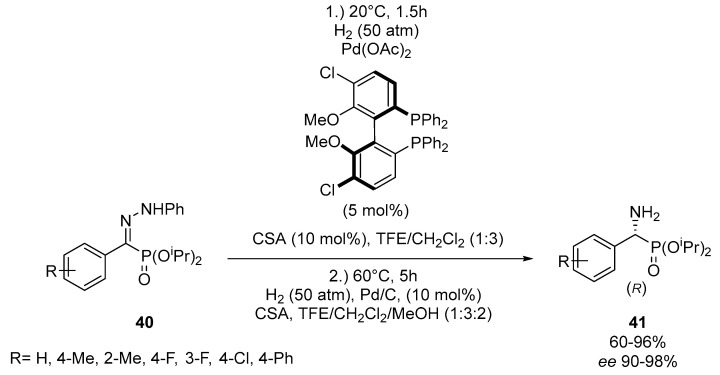
Pd-catalyzed asymmetric hydrogenation of α-hydrazonophosphonates (**40**).

The Pd-catalyzed asymmetric hydrogenation of α-hydrazonophosphonates (**40**) was performed under mild conditions. After a second, more forcing hydrogenation, the method provided the corresponding α-aminophosphonates (**41**) in up to 96% yield and 90–98% enantiomeric purities (Scheme 15) [27].

**Scheme 16:**
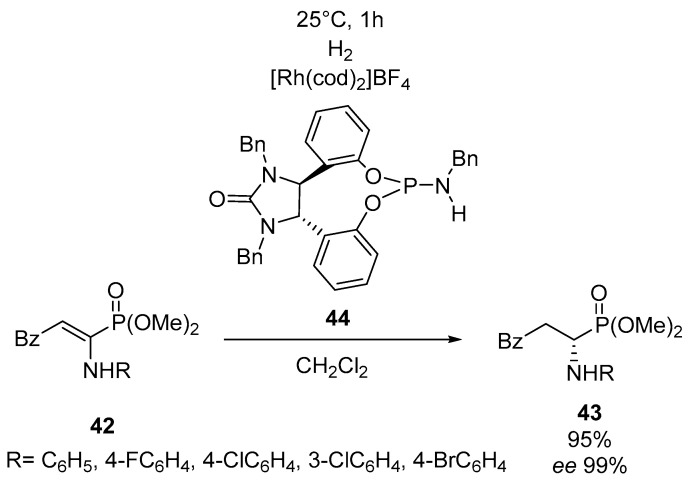
Asymmetric hydrogenation of α,β-enaminophosphonates (**42**) using a rhodium(I)-phosphoramidite (**44**) catalyst.

Asymmetric hydrogenation of α,β-enaminophosphonates (**42**) was performed using a rhodium(I)-phosphoramidite (**43**) catalyst (Scheme 16) [28]. This method gave the corresponding products (**44**) in up to 95% yield and 99% enantiomeric purity under mild reaction conditions.

**Scheme 17:**
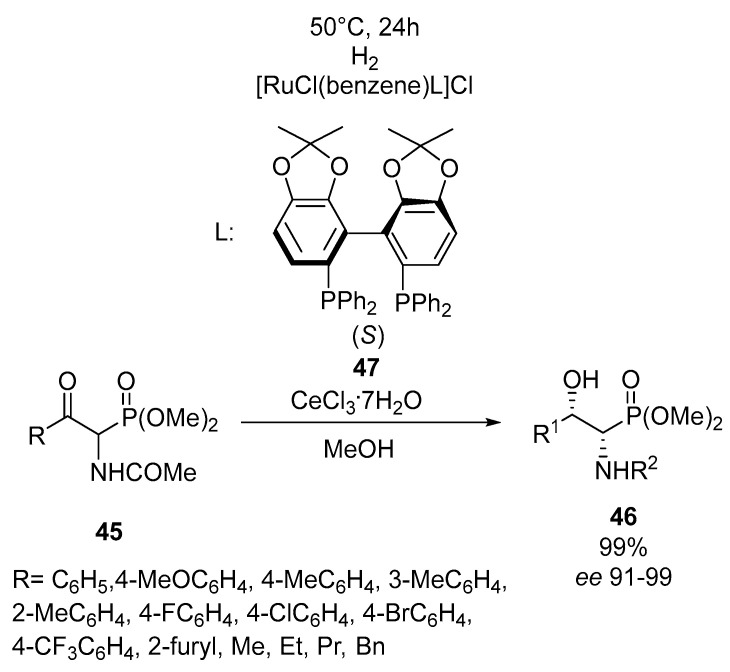
Asymmetric hydrogenation of α-acylamino-β-ketophosphonates (**45**).

α-Acylamino-β-ketophosphonates (**45**) were subjected to selective hydrogenation affording β-hydroxy-α-aminophosphonates (**46**) in high diastereo- and enantioselectivities. The conditions included a ruthenium chloride catalyst incorporating an optically active (S) bis(diphenyl-phosphino)biaryl ligand (**47**) (Scheme 17) [29].

In this sub-chapter, in most cases, enantiopure α-aminophosphonates were synthesized by asymmetric hydrogenation of iminophosphonates and enaminophosphonates. The methods discussed apply chiral catalysts, thereby allowing for the preparation of the corresponding products in good yields and 72–99% enantiomeric purities. The application of transition metal catalysts and the complexity and high cost of P-ligands constitutes a disadvantage.

### 2.4. Optically Active α-Aminophosphonates by Miscellenious Methods

**Scheme 18:**
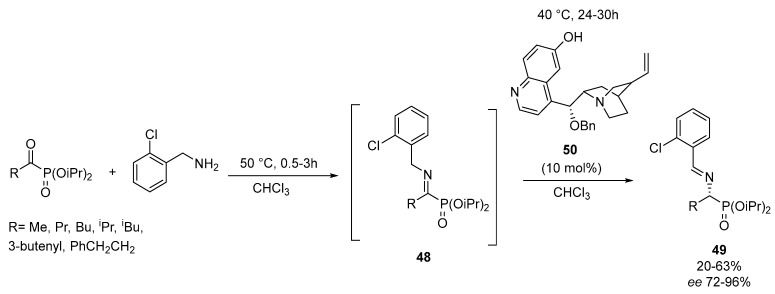
Asymmetric synthesis of α-iminophosphonates (**49**) by desymmetrization.

The asymmetric synthesis of α-iminophosphonates (**49**) was performed in two steps. First, the imin (**48**) was prepared, followed by a desymmetrized isomerization in the presence of a chiral cinchona catalyst (**50**). Following a deprotonation on the methylene unit of the benzyl group, an enantioselective protonation constituted the main enantio-differentiating step. The deprotonated species was complexed by the phenolic OH function of the cinchona catalyst, establishing a =N…H…O=P network. The yields of this elegant method were variable, and fell in the range of 20–63%. The enantiomeric purities were between 72 and 96% (Scheme 18) [30].

**Scheme 19:**
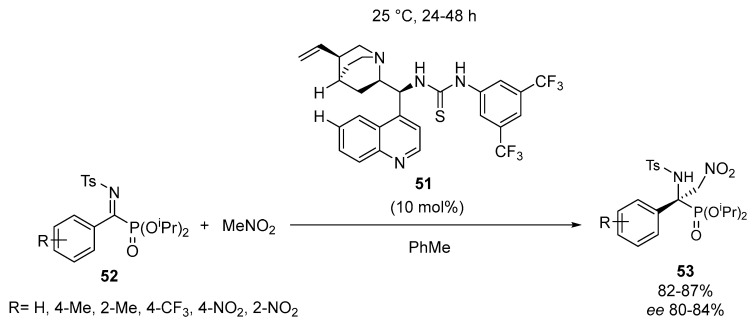
Asymmetric synthesis of α-aminophosphonates (**53**) by aza-Henry reaction.

A thiourea-coupled cinchona alkaloid-catalyzed (**51**) aza-Henry reaction of iminophosphonates (**52**) and nitromethane provided the corresponding α-aminophosphonates (**53**) under mild conditions in good yields, and with enantiomeric excesses (Scheme 19) [31]. This addition method has the advantage of 100% atomic efficiency.

**Scheme 20:**
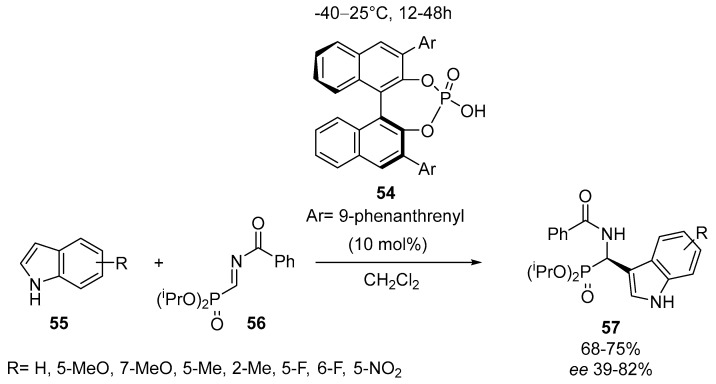
Enantioselective addition of indoles (**55**) to an iminophosphonate (**56**).

A chiral phosphoric acid (**54**) efficiently promoted the asymmetric addition of several indoles (**55**) to the N=C unit of α-iminophosphonate (**56)** to afford enantio-enriched α-aminophosphonate derivatives (**57**) (Scheme 20) [32].

**Scheme 21:**
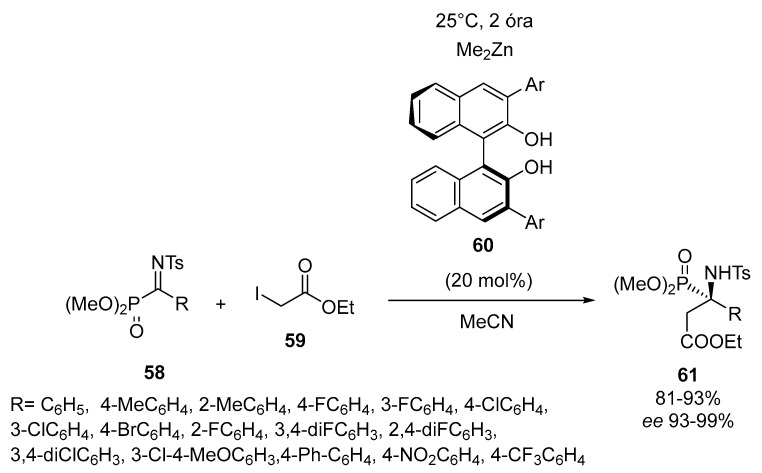
An enantioselective aza-Reformatsky reaction.

The Reformatsky synthesis is a valuable process that is widely used for the formation of C−C bonds. An enantioselective aza-Reformatsky reaction starting from iminophosphonates (**58**) was developed. The reaction of α-iminophosphonates (**58**) and iodoacetate (**59**) in the presence of a Zn catalyst with a BINOL-ligand (**60**) afforded the corresponding amino acid esters (**61**) in excellent yields and enantioselectivities (Scheme 21) [33]. The amino acid esters (**61**) were converted to amino acids, and then they were used in the synthesis of P-containing β-lactams. The products (**61**) were of high enantiopurity.

**Scheme 22:**
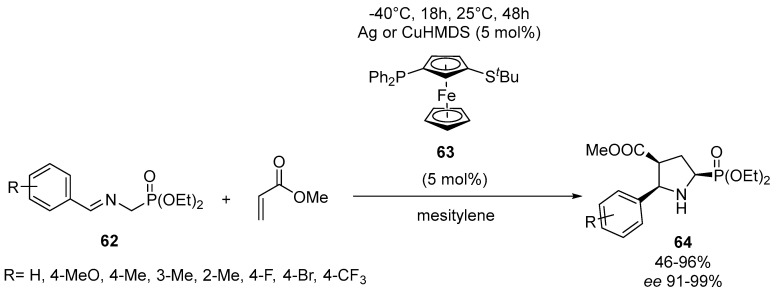
A catalytic asymmetric [3+2] cycloaddition reaction.

A catalytic asymmetric [3+2] cycloaddition reaction of iminophosphonates (**62**) with methyl acrylate carried out using a Cu- or Ag–FeSulphos catalyst (**63**) afforded phosphonoyl-proline derivatives (**64**) in variable yields and high enantioselectivities (Scheme 22) [34].

**Scheme 23:**
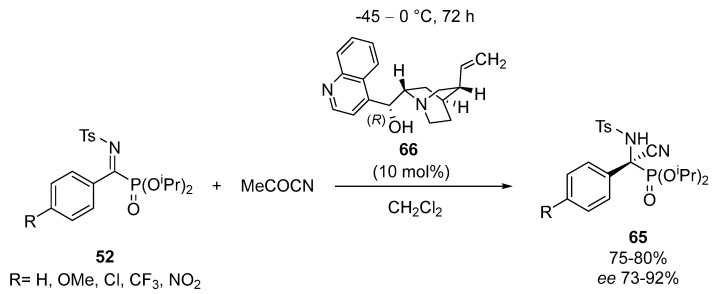
Asymmetric synthesis of α-amino-cianophosphonates (**65**) using cinchona alkaloid (**66**) as the catalyst.

α-Iminophosphonates (**52**) were converted to amino-cianophosphonates (**65**) in reaction with acetyl cyanid using a cinchona alkaloid (**66**) as the catalyst at −45–0°C. The yields fell in the range of 75–80%. The enantiomeric purity was 73–92% (Scheme 23) [35]. This transformation may be regarded as a special, rarely applied method. The low temperature needed is not robust.

**Scheme 24:**
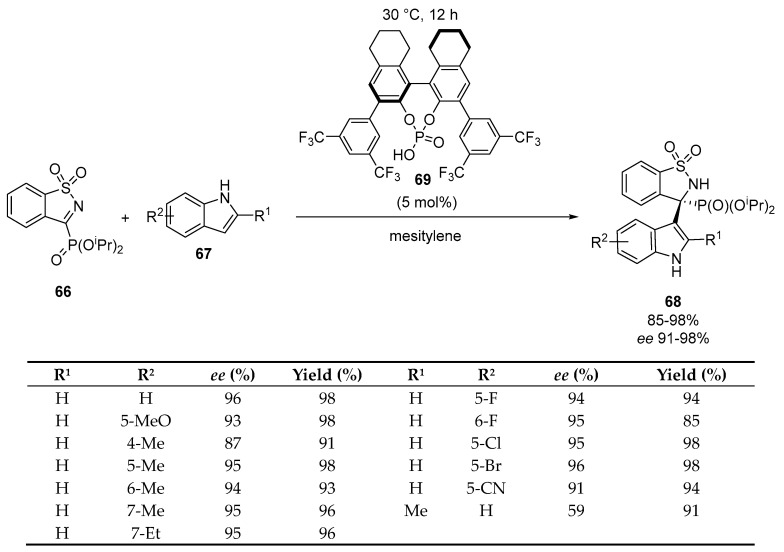
Enantioselective synthesis of indole-based α-aminophosphonates (**68**).

A series of optically active α-aminophosphonates (**68**) was synthesized from cyclic α-iminophosphonates (**66**) and indole derivatives (**67**) using a chiral phosphoric acid (**69**) as the catalyst under mild conditions. The method provided the corresponding multicyclic α-aminophosphonate derivatives (**68**) in 85–98% yields and 91–98% enantiomeric purities (Scheme 24) [36]. As this is an addition, the transformation is attractive due to its enantioselectivity.

**Scheme 25:**
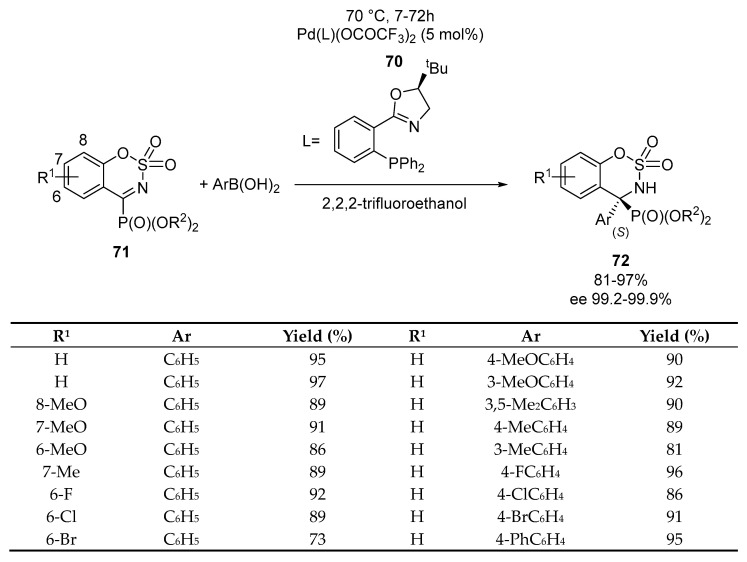
Enantioselective synthesis of cyclic α-aminophosphonates (**72**).

The Pd-phosphine complex (**70**)-catalyzed arylation of cyclic sulfonyl-iminophosphonate derivatives (**71**) with boronic acids gave the corresponding products (**72**) in 81–97% yields, and with up to 99% *ee* (Scheme 25) [37].

**Scheme 26:**
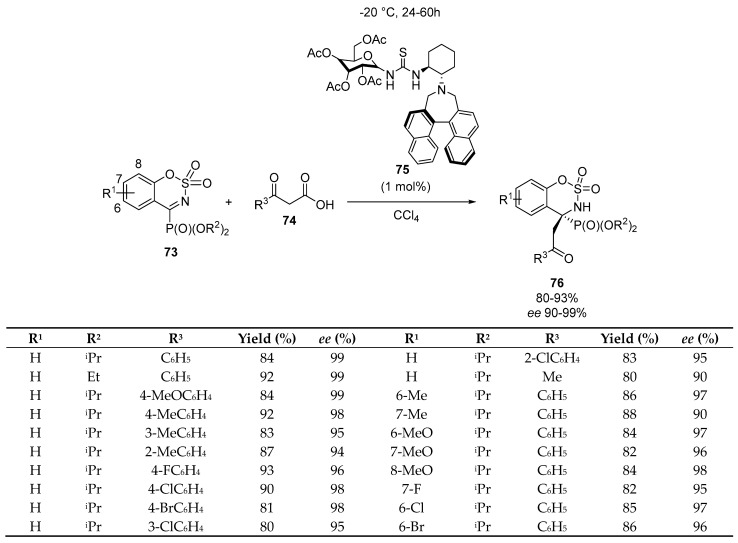
Saccharide-derived bifunctional amine-thiourea (**75**)-catalyzed Mannich reactions.

An asymmetric Mannich reaction was developed by reacting α-iminophosphonates (**73**) with keto acids (**74**) in the presence of a saccharide-derived bifunctional amine-thiourea organocatalyst (**75**). The yields were up to 93%, and the enantiomeric purities covered the range of 90–99% (Scheme 26) [38].

**Scheme 27:**
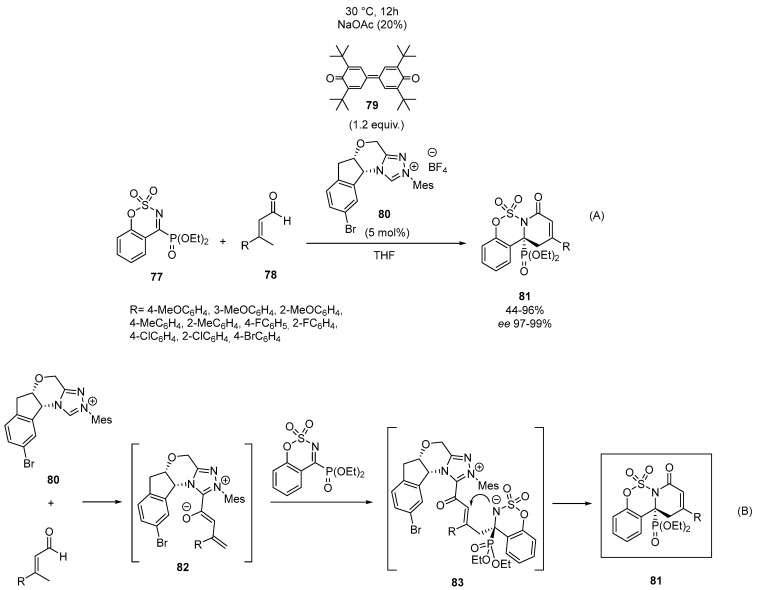
Enantioselective method for the synthesis of tricyclic α-amino phosphonates (**81**) (**A**), and the possible mechanism of the reaction (**B**).

The reaction of α,β-unsaturated aldehydes (**78**) and cyclic sulfonyl-iminophosphonate (**77**) in the presence of dibenzoquinone (**79**) as an oxidant afforded the corresponding products (**81**). The efficient carbene-catalyzed enantioselective cyclization reaction took place with 97–99% enantiomeric selectivity (Scheme 27) [39]. The precursor of the carbene is heterocycle **80**. The mechanism of the reaction was substantiated. The key steps involve the formation of a vinyl enolate intermediate (**82**) from enal and the catalyst, and the subsequent addition of vinyl enolate (**82**) to ketiminophosphonates to form the complex (**83**). The overall process is an asymmetric formal aza [4+2]-cycloaddition reaction.

Ordónez applied different *N*-acyliminium salts for the synthesis of racemic α-aminophosphonates [40].

**Scheme 28:**
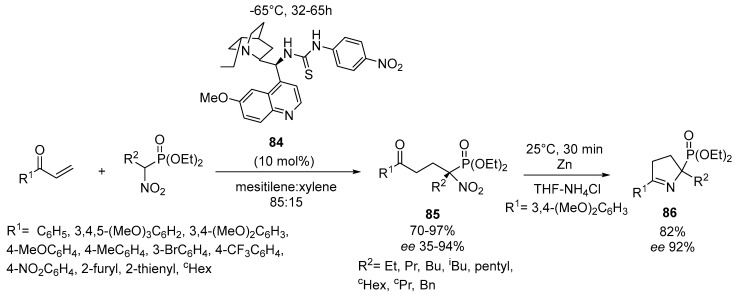
Synthesis of cyclic α-aminophosphonates (**86**) using a bifunctional organocatalyst (**84**).

The enantioselective Michael addition of α-nitrophosphonates to enones affording α-nitro-δ-oxophosphonates (**85**) was also described. Using a quinine thiourea chincona catalyst (**84**), the products (**85**) were obtained in good yields and variable enantioselectivities. The α-nitro-δ-oxophosphonates (**85**) were transformed to cyclic α-aminophosphonates (**86**) after the in situ reduction of the NO_2_ group, followed by intramolecular cyclization (Scheme 28) [41].

**Scheme 29:**
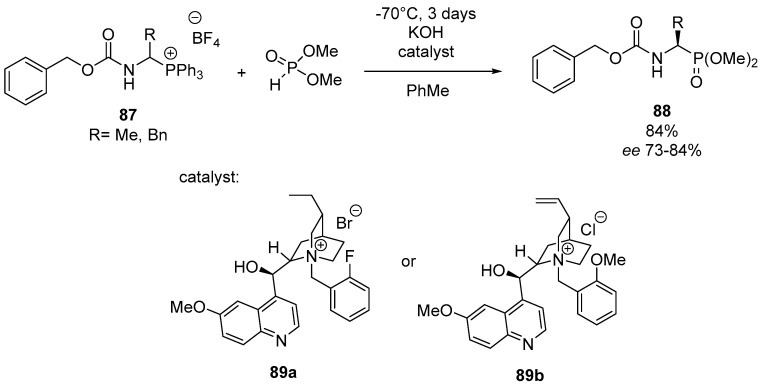
Enantioselective reaction of benzyloxycarbonylamino-alkylphosphonium salts (**87**) with dimethyl phosphite.

The reaction of benzyloxycarbonylamino-alkylphosphonium salts (**87**) with dimethyl phosphite in a cinchona-coupled quinine (**89a** or **89b**)-catalyzed method at −70°C led to optically active α-aminophosphonate derivatives (**88**) in good yields (Scheme 29) [42].

**Scheme 30:**
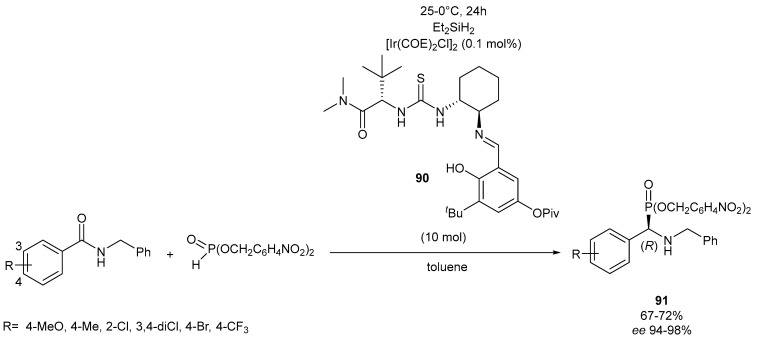
Enantioselective reductive phosphonylation of acetamide derivatives catalyzed by an iridium complex and a chiral thiourea organocatalyst (**90**).

Enantioselective reductive phosphonylation of acetamide derivatives using an iridium complex ([Ir(COE)_2_Cl]_2_: chlorobis(cyclooctene)iridium(I) dimer), and as a combination, a chiral thiourea organocatalyst (**90**) as the catalyst system, gave the optically active α-aminophosphonates (**91**) in good yields and high enantiomeric purities (Scheme 30) [43].

In this chapter, we collected methods for the synthesis of optically active α-aminophosphonates that can be obtained by different types of reactions. Each process results in the formation of the desired optically active product using a chiral catalyst. The methods presented, in some cases, may differ from each other. For example, they may differ in the reaction conditions used. A few protocols gave the corresponding optically active products either in good yields, or else only in good enantiomeric purity, but in lower yields. The selection of the best method for the synthesis the corresponding products depends on the substrates. The enantioselective methods demonstrated may be useful in special cases.

### 2.5. Synthesis of Enantioenriched α-Aminophosphonate Derivatives from Optically Active Starting Materials

**Scheme 31:**
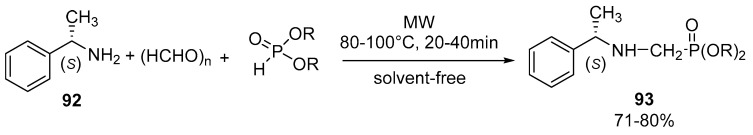
Synthesis of (*S*)-α-phenylethylamino-methylphosphonates (**93**) by Kabachnik–Fields reaction.

Dialkyl (*S*)-α-phenylethylamino-methylphosphonates (**93**) were synthesized from α-phenylethylamine (**92**), paraformaldehyde and dialkyl phosphites in an MW-assisted Kabachnik–Fields reaction without the use of any solvent. The yields fell in the range of 71–80%. During the reaction, the optical activity was retained (Scheme 31) [44,45].

**Scheme 32:**
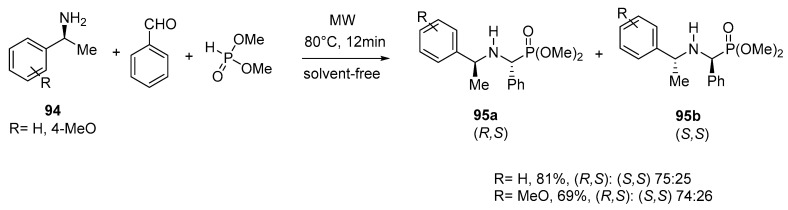
A diastereo-selective Kabachnik–Fields reaction.

In the MW-assisted solvent- and catalyst-free phospha-Mannich reaction of α-phenylethylamine derivatives (**94**) with benzaldehyde and dimethyl phosphite, the corresponding α-aminophosphonates (**95a** and **95b**) were formed in a diastereo-selective manner (Scheme 32) [46].

**Scheme 33:**
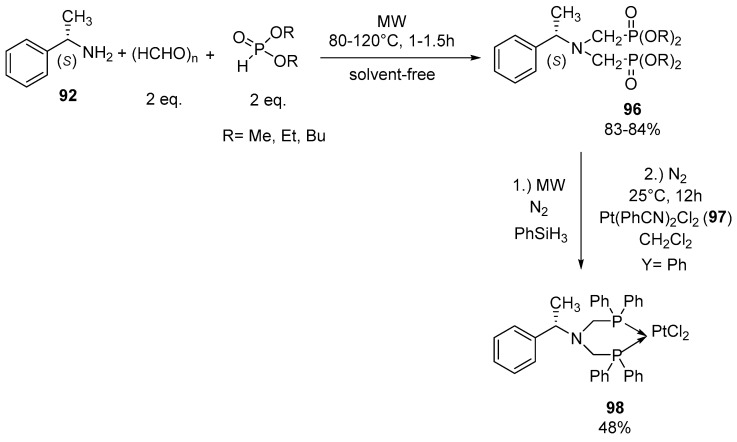
Synthesis of an optically active ring Pt complex (**98**).

Bis derivatives (**96**) could be synthetized in the reaction of (*S*)-α-phenylethylamine (**92**) with two equivalents of the oxo compound and two equivalents of the dialkyl phosphite. During the reaction, the optical activity was retained. The yields of the bis-α-aminophosphonate derivatives (**96**) were 83–84%. The (*S*)-bis(diphenylphosphinoylmethyl)-α-phenylethylamine (**96**) was double deoxygenated, applying phenylsilane under MW-assisted solvent-free conditions. Then, the optically active bisphosphine was converted to a chiral platinum(II) complex (**98**) by a reaction with dichlorodibenzonitrile platinum (**97**) (Scheme 33) [44,45].

**Scheme 34:**
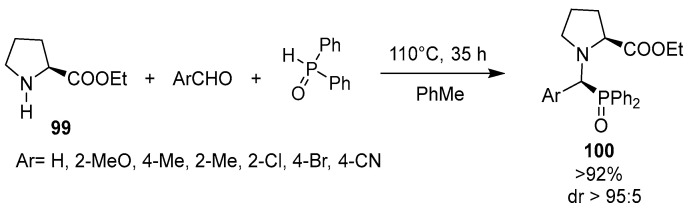
Synthesis of optically active α-aminophosphine oxides (**100**) from a proline ester.

Optically active α-aminophosphine oxides (**100**) were synthesized from the ethyl ester of chiral proline (**99**), benzaldehyde and diphenylphosphine oxide in toluene at reflux (Scheme 34) [47].

>P(O)H reagents derived from chiral alcohols were useful reagents in the preparation of optically active aminophosphonic derivatives [48].

**Scheme 35:**
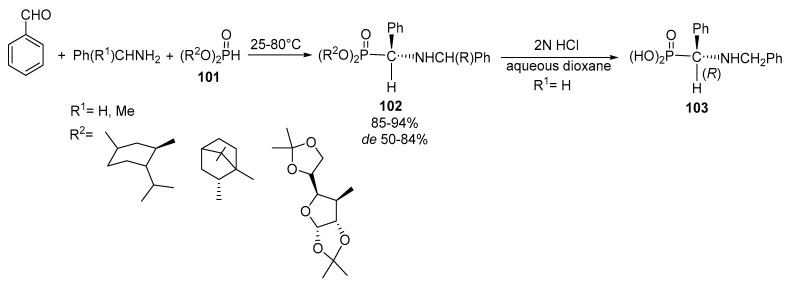
Kabachnik–Fields reaction with chiral dialkyl phosphites.

Chiral dialkyl phosphites (**101**) derived from (−)-borneol, (−)-menthol and (−)-1,2:5,6-di-O-isopropylidene-α-D-glucofuranose were applied as starting reagents for the preparation of chiral α-aminophosphonates (**102**) using the Kabachnik–Fields condensation. Good stereoselectivity was observed in the reactions. The transformation proceeded at room temperature, or upon heating (~60–80 °C) to give the corresponding α-aminophosphonates (**102**) in high yields and good stereoselectivities. Hydrolysis with 2 N HCl in aqueous dioxane gave the corresponding (*R*)-α-aminobenzylphosphonic acids (**103**) (Scheme 35) [49].

**Scheme 36:**
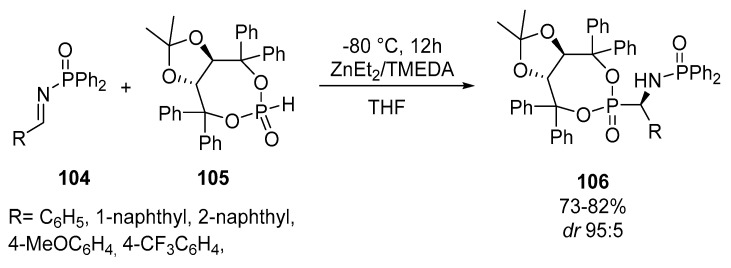
The synthesis of α-aminophosphonate derivatives (**106**) from the reaction of a phosphinoyl imine and (*R*,*R*)-TADDOL-based phosphite (**105**).

α-Aminophosphonic acid derivatives (**106**) were synthesized from *N*-diphenylphosphinoyl-imines (**104**) using a cyclic (*R*,*R*)-TADDOL-based phosphite (**105**) (Scheme 36) [50]. This is a good example for the application of a chiral phosphite.

α-Amido sulfones were suitable starting materials in the preparation of C-chiral α-aminophosphonic derivatives, using the above mentioned chiral phosphite (**105**) [51].

**Scheme 37:**
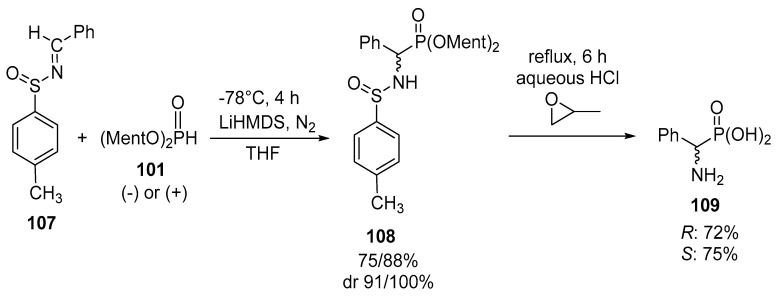
Asymmetric addition of chiral phosphites (**101**) to sulfinimines (**107**).

The addition of the two optically active dimenthyl phosphites (**101**, R^2^ = menthyl) to *N*-(*p*-tolylsulfinyl)-benzaldimine (**107**) afforded the corresponding sulfonyl-aminophosphonates (**108**) in a diastereomeric ratio of 91/100%. A subsequent acidic hydrolysis of the two sulfonyl-aminophosphonates (**108**) gave the corresponding optically active, free α-aminophosphonic acids (**109**) in 72/75% (Scheme 37) [52,53].

**Scheme 38:**
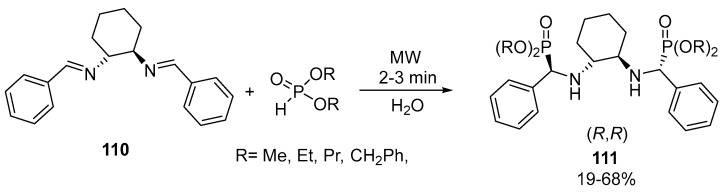
Synthesis of bis(aminophosphonates) (**111**) by the aza-Pudovik reaction.

1,2-cyclohexylenediamine-related bis(phenylmethylphosphonates) (**111**) were synthetized from the corresponding optically active bis-imine (**110**) and dialkyl phosphites in a diastereoselective manner without the use of any catalyst. The method afforded the corresponding bis(α-aminophosphonate) derivatives (**111**) under MW-assisted conditions in variable yields of 19–68% (Scheme 38) [54]. However, this procedure is not reproduceable due to the use of a kitchen MW oven.

The elegant methods shown above involve different optically active starting materials. The optical activity was preserved during the reactions. This kind of approach hides further future challenges. 

## 3. Conclusions

To summarize the contents of this review, the most useful methods for the synthesis of optically active α-aminophosphonates and related derivatives as described in the last decade were surveyed. The target compounds are important due to their potential and real biological activity. The most important approach is the asymmetric Kabachnik–Fields reaction of an oxo compound, an amine and a dialkyl phosphite performed in the presence of an optically active catalyst or additive. Another frequently used protocol is the enantioselective aza-Pudovik addition of >P(O)H regents to the C=N unit of imines in the presence of chiral catalysts. There were no data released on the possible re-circulation of the catalysts. Asymmetric catalytic hydrogenation of iminophosphonates is also an attractive synthetic method. It is an elegant approach if one of the components (e.g., the amine or the phosphite) of the Kabachnik–Fields condensation is optically active. Of course, there are special methods as well. Throughout the discussion, we tried to point out the “green” aspects, but also described the disadvantages.
